# Enhancing Trigger Point Dry Needling Safety by Ultrasound Skin-to-Rib Measurement: An inter-Rater Reliability Study

**DOI:** 10.3390/jcm9061958

**Published:** 2020-06-23

**Authors:** Anna Folli, Alessandro Schneebeli, Simone Ballerini, Francesca Mena, Emiliano Soldini, César Fernández-de-las-Peñas, Marco Barbero

**Affiliations:** 1 Rehabilitation Research Laboratory 2rLab, Department of Business Economics, Health and Social Care, University of Applied Sciences and Arts of Southern Switzerland, 6928 Manno, Switzerland; alessandro.schneebeli@supsi.ch (A.S.); marco.barbero@supsi.ch (M.B.); 2 Department of Business Economics, Health and Social Care, University of Applied Sciences and Arts of Southern Switzerland, 6928 Manno, Switzerland; simone.ballerini@supsi.ch; 3 Private physical therapy practitioner, 6850 Mendrisio, Switzerland; francesca.menafisioterapia@outlook.com; 4 Research Methodology Competence Centre, Department of Business Economics, Health and Social Care; University of Applied Sciences and Arts of Southern Switzerland, 6928 Manno, Switzerland; emiliano.soldini@supsi.ch; 5 Department of Physical Therapy, Occupational Therapy, Physical Medicine and Rehabilitation, Universidad Rey Juan Carlos (URJC), 28922 Alcorcón, Madrid, Spain; cesar.fernandez@urjc.es; 6 Cátedra Institucional en Docencia, Clínica e Investigación en Fisioterapia: Terapia Manual, Punción Seca y Ejercicio Terapéutico, Universidad Rey Juan Carlos, 28922 Alcorcón, Madrid, Spain

**Keywords:** trigger points, dry needling, adverse events, safety, skin-to-rib, reliability, ultrasound

## Abstract

Dry needling (DN) is a minimally invasive treatment technique widely used by physical therapists to treat myofascial trigger points (MTrP). Even if its safety has been commonly declared and the majority of adverse events are considered mild, serious adverse events cannot be excluded and DN treatments of several trunk muscles can potentially result in pneumothorax. Ultrasound imaging (US) skin-to-rib measurement could ensure the safety of this treatment procedure. Therefore, the aim of this study was to determine the inter-rater reliability of depth measurement of different trunk muscles (i.e., rhomboid, lower trapezius, iliocostalis, and pectoralis major) between an expert and two novice physiotherapists. Skin-to-rib distance of 26 asymptomatic and normal weights subjects was consecutively, independently, and randomly measured for each muscle by the three examiners (1 expert and 2 novice physical therapists) with a handheld US wireless probe. Intraclass correlation coefficient (ICC_3,k_) and standard error of measurement (SEM) were used to assess inter-rater reliability. Inter-rater reliability of skin-to-rib measurements between the three examiners was good to excellent or excellent for every muscle, with an ICC_3,k_ ranging from 0.92 and 0.98 (95% CI 0.86–0.99). The SEM never exceeded 10% of the skin-to-rib distance. In conclusion, skin-to-rib US measurements of the trunk muscles can be reliably performed by novice physical therapists using a handheld US device. These measures could be used as an innovative and reliable technique to improve the safety of some potential dangerous DN treatments.

## 1. Introduction

Trigger point dry needling (DN), as described in 1979 by Lewit [1] and popularized in the early 1990s by Hong [2], is a minimally invasive treatment technique that has become commonly used by physical therapists around the world, either alone or in combination with other treatments. A solid needle is introduced through the skin and the muscle to target myofascial trigger points (MTrP). The underlying mechanisms of DN include mechanical and neurophysiological effects trying to explain the inactivation of MTrPs [3,4]. Dry needling is widely used to treat MTrP in the cervical, thoracic or lumbar muscles associated with musculoskeletal conditions such as neck pain [5] and low back pain [6]. 

Since DN is an invasive intervention, its safety and the prevention of adverse events should be improved. Despite the commonly declared safety of DN, Brady et al. found that approximately 20% of the treatments (*n* = 7629) performed by physical therapists resulted in mild adverse events [7]. The most common adverse events were bruising (7.55%), bleeding (4.65%), pain during treatment (3.01%), and pain after treatment (2.19%). Importantly, no significant adverse events were reported giving an estimated risk rate of ≤0.04% of the treatment [7]. Similarly, a recent study also described that most adverse events of DN application were bleeding (16%), bruising (7.7%), and pain (5.9%), which were all categorized as minor [8]. Nevertheless, serious adverse events cannot be excluded. 

Although this skilled technique is considered safe if practiced by a well-trained practitioner [9], proper knowledge of human anatomy is needed for a safe application of DN [10,11]. One of the serious adverse events commonly discussed when DN is performed around the thoracic spine or rib cage [12] is the pneumothorax, “the entry of air into the pleural space” [13]. In fact, the literature describes some cases of pneumothorax during clinical practice [14,15] or teaching demonstration [16]. 

Two extensive literature reviews on the adverse events associated with acupuncture, including both primary and secondary reports, described, respectively, 715 and 1038 cases [17,18]. A total of 307 pneumothorax cases were reported retrospectively, and four cases were recorded in prospective surveys.

Even if the cases of pneumothorax reported in the literature following insertion of small solid gauge needles, as those used in DN or acupuncture are less than one in 100,000 patients treated [19], this is considered a serious adverse event and can require urgent treatment. 

Due to their anatomical proximity with the lungs, several muscles frequently treated by physical therapists with DN such as the mid or lower trapezius, rhomboid, pectoralis major, or iliocostalis can potentially result in pneumothorax. In some cases, a few authors have proposed imaging modalities as the only way to ensure the safety of the treatment procedure [11]. Ultrasound imaging could be an easy-to-use, low-cost, and radiation-free method to determine muscle thickness [20] and to avoid or decrease the occurrence of complication during therapeutic [21] or diagnostic [22] needle procedures. In fact, several studies have proposed different ultrasound (US) imaging protocols for assessing thickness or depth evaluation of several trunk muscles, e.g., rhomboids [23], pectoralis major [24], or lower trapezius [25], and have reported moderate to excellent relative inter- and intra-rater reliability associated with appropriate measurement errors, as well as agreement with other methods as MRI [26]. 

Nowadays, with technology development, portable US devices for evaluating the musculoskeletal system have emerged. To the best of the authors’ knowledge, no studies have previously investigated the reliability of depth measurement of different trunk muscles between novice physiotherapists and an expert clinician with a handheld US device. Proper identification of thorax musculature depth could assist clinicians in safer DN procedures of these muscles. Therefore, the aim of this study was to determine if a novice physiotherapist, after taking a crash course, can reliably conduct US skin-to-rib measurements over the pectoralis major, rhomboid, lower trapezius, and iliocostalis muscles on a sample healthy subjects aimed to improve DN safety. If the reliability of the skin-to-rib measurements by a novice physical therapist using a handheld US device is confirmed, there will be a basis for implementing ultrasound measures in the clinical practice of DN, as well as in education. This will support the decision-making on the depth of needling and will increase the safety of some risky DN techniques. 

## 2. Materials and Methods

The current cross-sectional inter-rater reliability study was conducted between October 2019 and February 2020 at the University of Applied Sciences and Arts of Southern Switzerland. The study was approved by the local ethics committee (CE 3438), and volunteers signed written informed consent forms prior to their participation.

### 2.1. Participants

A convenience sample was recruited via announcements in the institutional newsletters. A total of 26 asymptomatic and normal weights (BMI 18.5–24.9) subjects (50% male) were consecutively enrolled in the study. The following exclusion criteria were applied: 1, local skin lesions; 2, allergic reaction to US gel; 3, metal implants in the scanned area; 4, pacemaker implant; 5, inability to obtain testing positions. Sample size calculation was estimated according to Walter et al. [27] using the following parameters: three replicates, *p*0 = 0.6, *p*1 = 0.8, *α* = 0.05, and *β* = 0.2. The optimal sample size was 26 participants. 

### 2.2. Ultrasound Imaging Procedure

A handheld US wireless linear probe (128E Wireless Probe Ultrasound Scanner, SonoStar Technologies Co., Limited, Guangzhou, China) (frequency range 7.5–10 MHz) was connected with an 11.6 inch tablet (iPad, Apple Computer, Cupertino, CA, USA). An application (Wireless USG, SonoStar Technologies Co., Limited, Guangzhou, China) installed on the tablet was used to visualize the US images of the following muscles on both sides: rhomboid, lower trapezius, iliocostalis, and pectoralis major. 

For acquisition of pectoralis major muscle images, subjects laid supine on a treatment table, with both upper extremities by the sides and with a pillow under the head and neck. For acquisition of rhomboid, lower trapezius, and iliocostalis muscle images, subjects laid prone on a treatment table, with the face placed in the cutout of the plinth and both arms lying on the plinth arms support.

**Figure 1:**
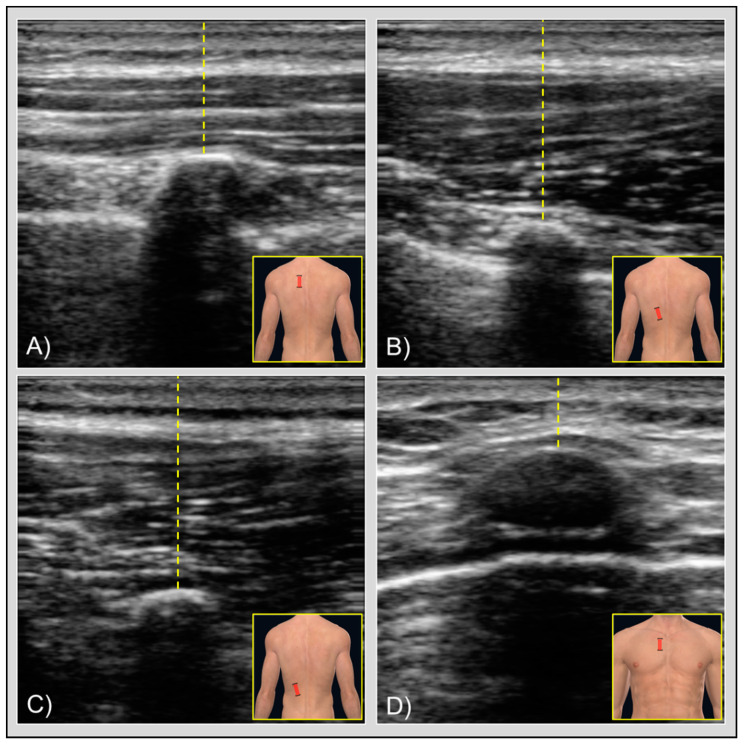
Figures showing the transducer placement (brackets) with ultrasound images and skin-to-rib measurement (yellow lines) for (**A**) rhomboid muscle, (**B**) lower trapezius muscle, (**C**) iliocostalis muscle, and (**d**) pectoralis major muscle. At the center of each image, the curved hyperechoic area represents the apex of the rib. Below the bone is visible the typical hypoechoic signal, also known as acoustic shadowing. Slightly deeper, in quadrants (**A**,**B**,**D**), you can also notice other hyperechoic tissues lateral to the bone, which represent the pleura. In the rhomboid muscle image (**A**), the different muscle layers are also visible: directly above the skin the middle trapezius muscle, just below it the rhomboid muscle, and around the rib the intercostal muscles. In the pectoralis major muscle image (**D**), the rib apex is less visible than in the other images due to the costal cartilages.

Before each experimental session, an external operator marked on the subject’s skin using a pen all the acquisition points. The following locations were assessed:

- Rhomboid (Figure 1A): halfway between the medial margin of the scapula and the spinous process of the spine, and halfway between the spine and the inferior angle of the scapula; transducer placed longitudinally.
- Lower trapezius (Figure 1B): 2–3 cm lateral to the spinous process of T8; transducer placed longitudinally.
- Iliocostalis (Figure 1C): 4–5 cm lateral to the spinous process of T10; transducer placed longitudinally.
- Pectoralis major (Figure 1D): lateral to the sternal origin and underneath the clavicular origin of the muscle; transducer placed transversally.

A final screening ensured that each selected acquisition point permitted to include in the US imaging at least one rib. The proposed acquisition points were introduced to replicate a potential location of a MTrP for the considered muscles.

### 2.3. Examiner Training

The US measurements were carried out by three examiners. Examiner 1 was a physiotherapist with 8 years of clinical practice, 7 years of US measurement experience, and 5 years of DN experience. Examiners 2 and 3 were two novice physiotherapists with 2 and 1 years of clinical practice, respectively and without US measurement experience nor DN previous education. Prior to experimental sessions, examiner 1 provided a US crash course to both examiners 2 and 3. The course was designed according to current education and included: 1, a brief US theoretical introduction; 2, an explanation of the US procedures to be used in the study, and 3, one hour and a half of practice with the US handheld device. The contents were presented through a frontal lecture with the projection of slides. Examiner 2 and 3 could at any time interrupt the contribution and ask questions of clarification or deepening. The practical part was carried out simultaneously by both examiners, under the supervision of the skilled experienced examiner. During this phase, asking questions or for further explanations was also allowed.

### 2.4. Image Capturing Assessment

All participants were consecutively, independently, and randomly examined by the three examiners, without changing their position during the examination session. Examiners were asked to provide a small amount of pressure with the US probe during the measurement to mimick the DN technique where the therapist’s fingers apply a slight pressure on the muscle harboring the MTrP. This ensured an appropriate thickness measurement when considering the DN practice as well as its safety. Handling of the probe and pressure on the target muscle was instructed and trained during the crash course.

Every examiner acquired the image and immediately took the skin-to-rib distance (in mm) for each image, measuring the shortest linear distance from the skin to the more cranial rib, directly on the tablet that was connected to the US transducer (Figure 1). This procedure was repeated 3 times for each point, and the mean of the 3 measures was considered for further statistical analyses. This process took around 2.5 min on each point. The area of the tablet displaying the numerical thickness measurement was masked by a sticker so that the examiners were blinded to their own previous measurements. An external operator stored the measurements, ensuring the blinding of the examiners. Examiners were blinded to the findings of other examiners during the study.

### 2.5. Statistical Analysis 

Data analyses were carried out using the Statistical Package for Social Sciences for Windows (SPSS, Chicago, IL, USA), version 26. Descriptive statistics (mean and standard deviations) were used to describe the sample characteristics (i.e., age, gender, BMI) and to report the skin-to-rib measures of each muscle. Intraclass correlation coefficient (ICC) and their 95% confidence intervals (95% CIs) were calculated to assess inter-rater reliability between the three examiners on each selected muscle, based on a mean-rating (*k* = 3), absolute agreement, 2-way mixed-effects model (ICC_3,k_). The criteria used for the interpretation of the ICCs were according to Koo and Li [28]: 0.00–0.5: poor; 0.5–0.75: moderate; 0.75–0.9 good; 0.9–1.0 excellent. ICC values higher than 0.6 were considered clinically relevant [29]. Standard error of measurement (SEM) was also calculated to assess absolute reliability (i.e., SEM = s_x_$\sqrt{1-\mathrm{ICC}}$, where s_x_ is the standard deviation of the measures acquired on the analyzed point). 

## 3. Results

A total of 26 subjects (50% males) were evaluated with US by three examiners (two novice and one expert) on the pectoralis major, rhomboid, lower trapezius, and iliocostalis muscles, repeating each evaluation three times (24 images per examiner per subject, 468 images per muscle, 1872 images overall). 

The mean (± SD) age, height, weight, and BMI of the participants were 28 ± 7 years old, 1.7 ± 0.1 cm, 65.4 ± 9.4 kg, and 21.9 ± 2.0 kg/m^2^, respectively (Table 1).

Inter-rater reliability of skin-to-rib measurements between Expert and Novice 1, Expert and Novice 2, and Novice 1 and Novice 2 examiners was good to excellent for every muscle, with an ICC_3,k_ ranging from 0.92 to 0.98 (95% CI 0.86–0.99). The SEM never exceeded 10% of the depth of the rib: pectoralis major (SEM 0.46–0.68 mm, 4.9–7.3%); rhomboid (SEM 0.91–0.94 mm; 5.6–5.8%), lower trapezius (SEM 1.25–1.45 mm, 5.45–6.3%), and iliocostalis (SEM 0.90–1.10 mm; 4.25–5.15%). Table 2 shows reliability data for each muscle for the three examiners.

## 4. Discussion

The main aim of this study was to assess the inter-rater reliability of US measurements with a handheld US device made by three examiners considering their experience. We hypothesized that a novice examiner could reliably perform US skin-to-rib measurements using a handheld US device as compared to an experienced clinician. Despite the difference in the experience in DN and US imaging of the three examiners, the results of our study revealed good to excellent inter-rater reliability skin-to-rib measurements on the rhomboid, lower trapezius, iliocostalis, and pectoralis major muscles. Additionally, the observed SEM never exceeded 10% and was maximum about 1.5 mm. When considering DN practice in the thorax region for the target muscles, the safety procedures will be affected by measurement errors about millimeters.

Two previous studies have analyzed the skin-to-rib distance of rhomboid muscle to determine the appropriate depth of needle insertion during DN treatment and the influence on these distances of positional changes, body composition, and sex of the subject [30,31]. Mitchell et al. [30] analyzed 60 subjects (20 subjects in each of the three BMI groups—normal weight, overweight, and obese) at the level of levator scapulae, rhomboid minor, and rhomboid major. These authors found that the rib depth at the level of rhomboid major in normal-weight subjects was 1.7 cm (±0.3 cm) at the level of the fifth rib and 1.5 cm (±0.3 cm) at the level of the sixth rib without bolster; and 2.2 cm (±0.5 cm) at the level of the fifth rib and 2.0 (±0.5 cm) at the level of the sixth rib with bolster. In the current study, we found a mean skin-to-rib distance of 1.6 cm (±0.3 cm) on the rhomboid muscle, which agrees with Mitchell et al.’s study [30]. We cannot be sure of the exact rib level of our acquisition, but we estimate that we were in between the fourth and the seventh rib. Nevertheless, there are some differences between the two imaging protocols (i.e., the subjects in our study were placed with arms on the plinth support and the examiners applied a pressure similar to that exerted during DN treatment), which may explain the small difference (0.1 cm) between studies. Additionally, this study also reported excellent (ICC 0.997) intra-rater reliability in the skin-to-rib measurement [30]. These results are also consistent with our reliability data, showing ICC > of 0.8 for the rhomboid muscles for the different examiners. However, we should consider that Mitchell et al. [30] investigated intra-rater reliability, whereas we assessed inter-examiner reliability. Seol et al. [31] measured the skin-to-rib distance on 62 patients at the level of rhomboid muscle. Their BMI categories were slightly different from ours, but their classes corresponding to our “normal weight” were found to have a skin-to-rib of 2.1 cm (±0.4 cm) and 2.4 cm (±0.9 cm). The difference between their and our data could be attributed to the differences in the methodology. Even if the acquisition point on the image is more similar to that of Mitchell and al. [30], participants were seated with the hand on the opposite shoulder and not prone. Moreover, also in the study of Seol et al. [31], no pressure was exerted with the US probe. The discrepancies between the skin-to-rib of Seol et al. [31] and our study underline the importance to have the chance to measure in each situation the depth of the rib and to profit from this useful information for the clinical judgment in the treatment of patients with needling procedures. Finally, our study is the first one investigating inter-rater reliability considering the experience of the assessor, which increases the applicability of our results in clinical practice. 

Intra-rater reliability was not evaluated in our study for a few reasons. First, because high intra-rater and test–retest reliability of US muscle thickness measurement have already been demonstrated in the trunk muscles [20,23,30,32] as well as in other body regions [33,34,35,36]. Secondly, during semi-controlled experiments prior to the study, a high consistency was observed for the repeated US measurements among the different examiners. This is also supported by the very low observed SD measurements in this study.

Positional modifications, sex, and BMI of the subject led to important changes in tissue depth and muscles thickness [10,20,30,31]. Therefore, US measurement assessing the real distance between the skin and potential dangerous-to-reach anatomical structures can enhance the DN safety when treating MTrPs on thoracic musculature. The skin-to-rib US measure can be used as an indication for a safe needle insertion depth or as a cue to select the proper needle size. Since the measure is reliable, a clinician may choose the shortest needle to reach the targeted muscle and to not overcome the ribs depth, reducing the potential risk of pneumothorax, especially for novice therapists with DN technique. Current and previous data [30,31] suggest that the needle should not be inserted deeper than 1.5 cm for reaching the rhomboid and not more than 2 cm for reaching the lower trapezius and iliocostalis muscles in normal-weight patients. Nevertheless, it is important to consider that manual palpation of the ribs over the rhomboid muscle has shown a moderate accuracy (66.3%) rate [21]; therefore, any DN intervention over the rib cage should be conducted with caution for avoiding pneumothorax. Similarly, based on our results, the needle should not be inserted deeper than 0.9 cm for reaching the pectoralis major muscle, the clavicular portion, although further studies are needed in this muscle.

These US measures could be useful also during DN education, where students complain about the difficulty in estimating the skin-to-rib distance and the fear of running into adverse effects by inserting the needle into the thorax. In addition to inexperienced physiotherapists, US measures could be useful to any clinician facing doubtful situations such as patients with very high or low BMI, spinal deviations (i.e., scoliosis, hyper-kyphosis) or when the patient position during DN has to be modified for concurrent reasons. Nevertheless, these measurements are specific and only related to DN treatments. This may not justify the use of US within the physiotherapist practice, but the growing interest for rehabilitative ultrasound imaging can help to increase the meaningful US applications for physical therapists. Finally, it should be noted that physiotherapists acquire a solid knowledge of the musculoskeletal anatomy during their entry-level education and this may explain at least partially the excellent results obtained in the current study. The requested US application included superficial structures easy to be detected. Ultrasound-guided interventions or diagnostic procedures would require more extensive training and different education, and any generalization to different US applications has to be done cautiously. Further research is needed about the perceived usefulness and applicability in the clinical practice of the proposed procedures and about its efficacy in increasing the DN safety.

### Limitations

There are some limitations to consider in the current study. First, the sample chosen considered only normal-weight subjects. Measures could be more complex to take in different situations, for example in overweight subjects or women with particularly big chests (for pectoralis major muscle). 

Another potential limitation could be the pressure with the US probe, which was not controlled. Even if examiners were instructed to the reasonable amount of pressure, the variation in pressure between the examiners could have led to important between-examiner differences in the measures. Without pressure, the inter-rater reliability could have been even higher and our depth data could have been more comparable with those of previous studies. However, it is important to note that clinical application of DN usually involves slightly pressure of the superficial tissues over the MTrP taut band. Despite this, our results show high reliability between examiners. 

Another possible limitation of our study is that we acquired and analyzed the images without controlling the breath of the subjects; however, participants were relaxed on an examination table, and therefore a deep inspiration or expiration breath was unlikely. Finally, it should be acknowledged that only healthy volunteers have been enrolled in this study. Although we did not identify any critical elements associated with patients complaining of MTrPs that may limit the application of US skin-to-rib measurements, caution should be used in generalizing our findings to different populations.

## 5. Conclusions

Our results indicate that after a crash course, skin-to-rib US measurements of the rhomboid, lower trapezius, iliocostalis, and pectoralis major muscle can be reliably performed by a novice physical therapist using a handheld US device. The clinical relevance of the proposed skin-to-rib US measurements remains unknown, but this study provides the basis to introduce these measures as an innovative and reliable method to manage the safety of some potential dangerous DN treatments.

## Figures and Tables

**Table 1:** Demographic and ultrasound data of the total sample.

Subjects	Age (Years)	Height (m)	Weight (kg)	BMI (kg/m^2^)	Thickness (mm)			
All (*n* = 26)	28 (7)	1.72 (0.09)	65.4 (9.4)	21.9 (2.0)	9.3 (2.5)	16.3 (3.4)	22.9 (5.4)	22.0 (6.6)
Men (*n* = 13)	29 (8)	1.79 (0.06)	72.4 (5.7)	22.6 (1.7)	8.8 (2.1)	17.2 (2.7)	25.4 (4.5)	23.9 (6.3)
Women (*n* = 13)	26 (4)	1.66 (0.06)	58.3 (6.6)	21.3 (2.2)	9.9 (2.7)	15.3 (3.7)	20.4 (5.1)	20.1 (6.4)

**Table 2:** Inter-rater reliability (ICC_3,k_, 95% CI, SEM) of thickness measurements based on US acquisitions on different muscles of three examiners.

Muscle	Mean (±SD) (mm)	Expert-Novice 1	Expert-Novice 2	Novice 1-Novice 2
Pectoralis major	Expert: 9.3 (2.5) Novice 1: 9.5 (2.6) Novice 2: 9.3 (2.4)	0.967, 95% CI 0.943–0.981, SEM: 0.46	0.959, 95% CI 0.929–0.977, SEM: 0.49	0.926, 95% CI 0.871–0.957, SEM: 0.68
Rhomboid	Expert: 16.3 (3.4) Novice 1: 15.9 (3.2) Novice 2: 16.2 (3.3)	0.923, 95% CI 0.866–0.956, SEM: 0.91	0.920, 95% CI 0.861–0.954, SEM: 0.94	0.919, 95% CI 0.859–0.953, SEM: 0.93
Lower trapezius	Expert: 22.9 (5.4) Novice 1: 22.2 (5.0) Novice 2: 22.5 (4.8)	0.921, 95% CI 0.861–0.955, SEM: 1.45	0.939, 95% CI 0.894–0.965, SEM: 1.25	0.928, 95% CI 0.875–0.958, SEM: 1.30
Iliocostalis	Expert: 22.0 (6.6) Novice 1: 21.0 (6.3) Novice 2: 21.3 (6.6)	0.969, 95% CI 0.928–0.985, SEM: 1.13	0.980, 95% CI 0.960–0.990, SEM: 0.93	0.973, 95% CI 0.954–0.985, SEM: 1.06

ICC: intra-class correlation coefficient; SEM: standard error of measurement
